# Complement C4d-specific antibodies for the diagnosis of lung cancer

**DOI:** 10.18632/oncotarget.23690

**Published:** 2017-12-26

**Authors:** Daniel Ajona, Marcin Okrój, María J. Pajares, Jackeline Agorreta, María D. Lozano, Javier J. Zulueta, Carla Verri, Luca Roz, Gabriella Sozzi, Ugo Pastorino, Pierre P. Massion, Luis M. Montuenga, Anna M. Blom, Ruben Pio

**Affiliations:** ^1^ Program in Solid Tumors, Center for Applied Medical Research (CIMA), CIBERONC, and Navarra’s Health Research Institute (IDISNA), Pamplona, Spain; ^2^ Department of Biochemistry and Genetics, School of Sciences, University of Navarra, Pamplona, Spain; ^3^ Lund University, Department of Translational Medicine, Section of Medical Protein Chemistry, Malmö, Sweden; ^4^ Department of Medical Biotechnology, Intercollegiate Faculty of Biotechnology, University of Gdańsk and Medical University of Gdańsk, Poland; ^5^ Department of Histology and Pathology, School of Medicine, University of Navarra, Pamplona, Spain; ^6^ Department of Pathology, Clínica Universidad de Navarra, Pamplona, Spain; ^7^ Department of Pulmonary Medicine, Clínica Universidad de Navarra, Pamplona, Spain; ^8^ Tumor Genomics Unit, Department of Experimental Oncology and Molecular Medicine, Fondazione IRCCS Istituto Nazionale dei Tumori, Milan, Italy; ^9^ Thoracic Surgery Unit, Department of Surgery, Fondazione IRCCS Istituto Nazionale dei Tumori, Milan, Italy; ^10^ Thoracic Program, Vanderbilt Ingram Cancer Center, Vanderbilt University Medical Center, Nashville, USA

**Keywords:** lung cancer, diagnosis, biomarker, complement C4d, indeterminate pulmonary nodules

## Abstract

Development of molecular markers that help to identify high-risk individuals or diagnose indeterminate pulmonary nodules could have a major impact on lung cancer clinical management. In this study, we evaluated the diagnostic potential of a newly-developed ELISA that specifically detects complement C4d. We measured this marker in five independent cohorts of plasma and bronchoalveolar lavage samples from lung cancer patients and controls. In case-control studies, the area under the ROC curve for the diagnosis of lung cancer was 0.82 (95%CI = 0.72–0.92) in plasma samples, and 0.80 (95%CI = 0.69 to 0.90) in bronchoalveolar lavage fluids. In a set of plasma samples from the MILD CT-screening trial, the assay was unable to discriminate between asymptomatic high-risk individuals with or without early stage lung cancer. On the contrary, in two independent cohorts of individuals with indeterminate pulmonary nodules, plasma samples from patients with lung cancer nodules presented higher levels of C4d than those from patients with benign nodules. Using a target population of patients with 8 to 30 mm nodules, the test identified likely benign lung nodules with 84% negative predictive value and 54% positive predictive value, at 89% specificity and 44% sensitivity. In conclusion, the specific determination of C4d may serve as an adjunct to current clinical practice in the diagnosis of indeterminate pulmonary nodules.

## INTRODUCTION

Lung cancer is the leading cause of cancer death worldwide [1] and it is difficult to diagnose, particularly in its early stages, when survival rates are higher [2]. However, in the recent years, screening studies using low-dose computed tomography (CT) have reported high rates of lung cancer detection in early stages [3]. The National Lung Screening Trial (NLST), which included more than 50,000 participants, concluded that screening with the use of CT detects lung tumors at early stages (mostly stage I) and reduces lung cancer mortality by at least 20% [4]. Although lung cancer detection rates in CT screening programs are high [4–5], there are still many aspects of this strategy that require optimization. In this regard, molecular biomarkers may be helpful for the selection of those individuals with high risk of developing lung cancer. Besides, radiological techniques could be combined with molecular markers to determine which pulmonary nodules detected by CT are more likely to be malignant, and therefore require closer follow-up.

Several noninvasive molecular tests have been developed to evaluate the presence of lung cancer in asymptomatic persons, or to determine whether screening-detected indeterminate lung nodules are malignant [6–7]. These tests include the detection of miRNA-based markers [8–9], epigenetic and genetic markers [10–11], proteomic markers [12–13], or autoantibodies [14]. We have recently reported that C4d-containing fragments of complement C4 are elevated in plasma and bronchoalveolar lavage samples from lung cancer patients, and may be of value for the diagnosis of the disease [15–16]. Moreover, this marker may also have diagnostic value in head and neck malignancies [17]. C4d-containing fragments are generated from complement C4 upon activation of the classical pathway of complement, a central humoral component of innate immunity. Activation of C4 is achieved by the proteolytic cleavage of a single peptide bond, which converts C4 into C4b and C4a. C4b is the larger cleavage product and can bind to the target. C4b is subsequently cleaved and inactivated by factor I, which converts C4b into C4d through the intermediate product iC4b [18]. C4d is thus the final breakdown product of activated C4.

In our previous studies, a commercial ELISA was used for the quantification of C4d [15–16]. This assay measures C4d-containing fragments of complement C4, which comprises at least three elements: C4b, iC4b and C4d. The mixed determination of these fragments may affect negatively to the performance and clinical applicability of the diagnostic test. Therefore, in an attempt to develop an assay less prone to generate false positives, we developed an ELISA based on antibodies reactive to a short linear neoepitope with a high specificity for the C4d fragment [19]. In the present study we compared the diagnostic performance of the commercial assay and the newly designed tool in both plasma and bronchoalveolar lavage samples from lung cancer patients. Next, we evaluated the potential utility of the new assay in two clinically relevant contexts: identification of asymptomatic high risk individuals enrolled in a CT-screening program and evaluation of individuals with indeterminate lung nodules.

## RESULTS

### Specific quantitation of C4d outperforms the diagnostic accuracy of quantification of C4-derived fragments in lung cancer patients

**Figure 1:**
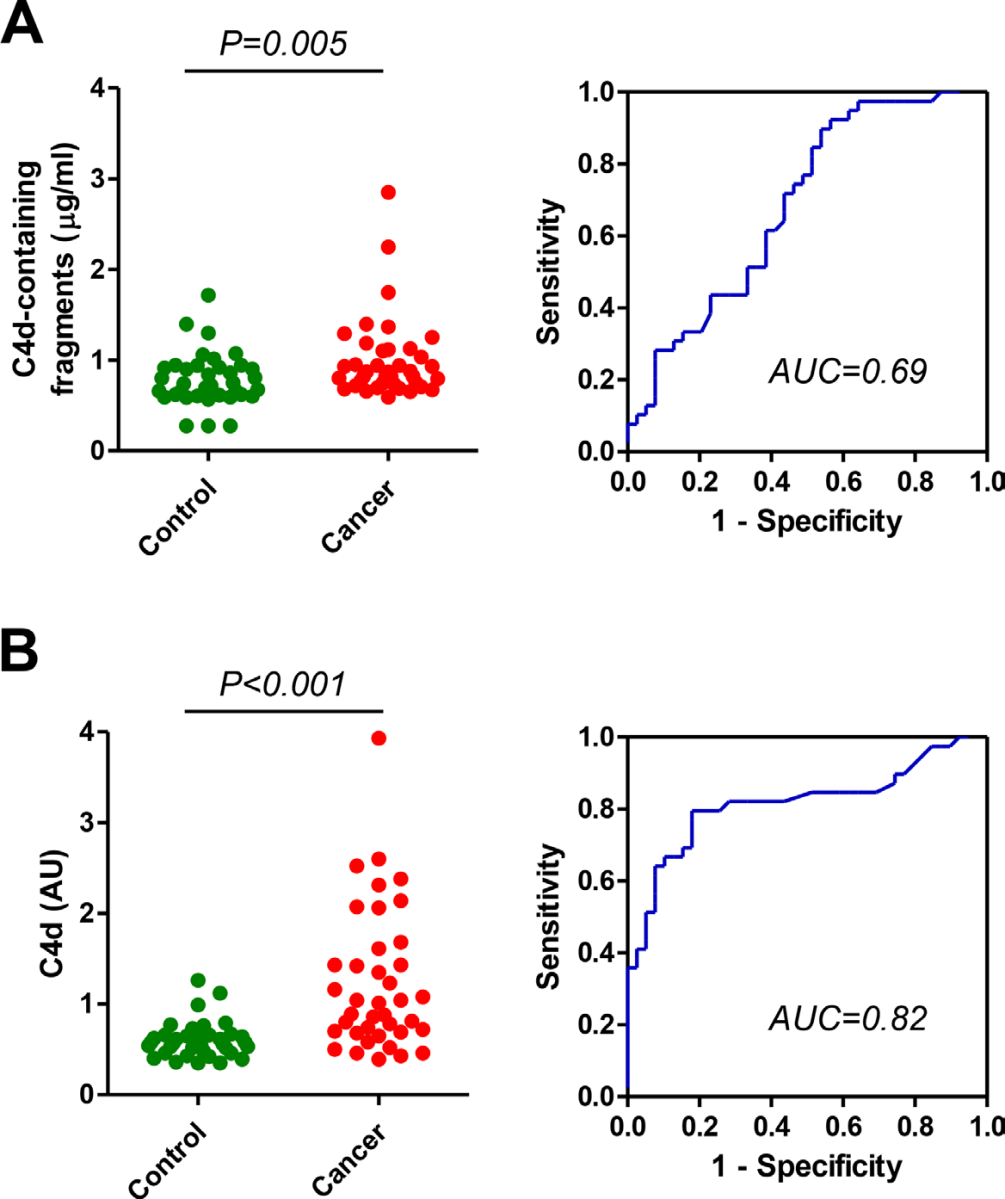
Quantitation of C4d-containing fragments and C4d in plasma samples from lung cancer patients and matched control subjects (**A**) Levels of plasma C4d-containing fragment in patients diagnosed with early stage (I and II) non-small cell lung cancer and control subjects. The area under the ROC curve was 0.69 (95% CI: 0.57-0.80). (**B**) Plasma C4d levels measured by highly specific antibodies in the same cohort of plasma samples. The area under the curve was 0.82 (95% CI: 0.72-0.92). *P* values were calculated using the two-sided Mann-Whitney *U*-test. AU: Arbitrary units.

**Figure 2:**
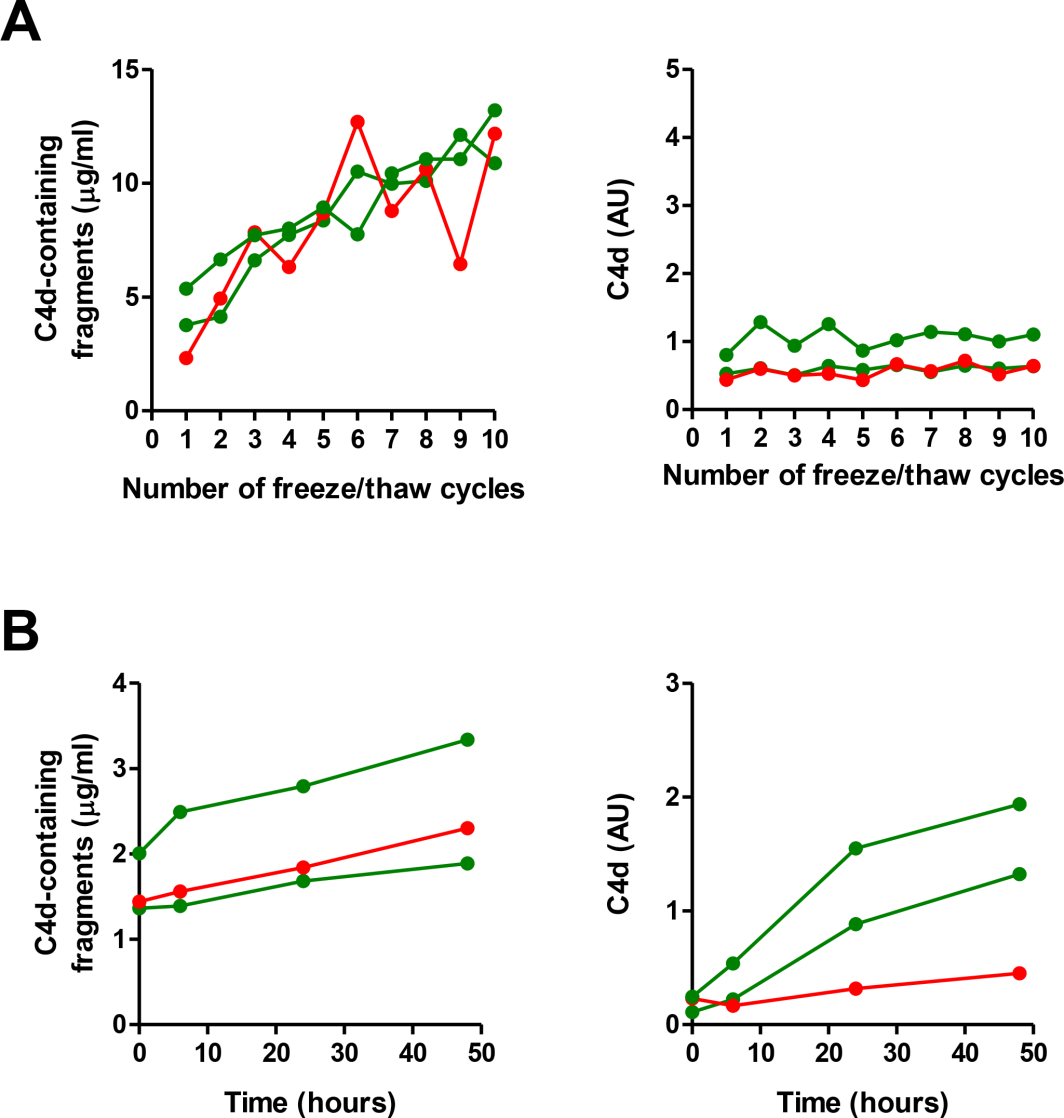
Impact of freezing/thaw cycles and incubation time in the quantitation of C4d-containing fragments and C4d Plasma levels of C4d-containg fragments and C4d were determined after 1 to 10 freezing/thawing cycles (**A**) or after incubation at room temperature up to 48 hours (**B**). These experiments were performed using plasma samples from two lung cancer patients and one control subject. AU: Arbitrary units.

In previous studies we found a significant elevation of proteolytic fragments of complement C4 in biological fluids from lung cancer patients using a commercial ELISA based on the detection of C4d-containing fragments (C4b, iC4b and C4d) [15–16]. Here we sought to evaluate the diagnostic performance of an assay based on highly specific antibodies against C4d, the end degradation product of activated C4. We initially compared the diagnostic accuracy of both assays in plasma samples from early stage (I or II) non-small cell lung cancer patients (*n* = 39) and control individuals (*n* = 39), matched by age, sex, and smoking status. Samples were obtained at Clinica Universidad de Navarra. In agreement with our previous studies, plasma samples from patients with early stage lung cancer showed significantly higher levels of C4d-containing fragments than plasma samples from control subjects (Figure 1A). Marker levels in patients and controls, expressed as median (interquartile range), were: 0.87 (0.74–1.12) and 0.72 (0.61–0.92) µg/ml, respectively (*P* = 0.005). The area under the ROC curve was 0.69 (95%CI = 0.57–0.80). The specific detection of C4d with the new antibodies improved the diagnostic performance of the test: 1.01 (0.69–1.61) and 0.58 (0.48–0.66) AU for lung cancer and control subjects, respectively (*P* < 0.001; Figure 1B). The area under the ROC curve was 0.82 (95% CI = 0.72–0.92). No association was found between C4d levels and epidemiological and clinical characteristics such as sex, age, smoking status, histology and stage (Supplementary Table 1). A weak positive correlation was found between the levels of C4d-containing fragments and the levels of C4d (Spearman’s rho = 0.286, *P* = 0.011; Supplementary Figure 1A). We also assessed the effect of handling conditions using plasma samples from two lung cancer patients and a control individual. Freezing/thawing cycles increased the plasma levels of C4d-containing fragments (Figure 2A). In contrast, the specific measurement of C4d was stable up to as many as ten freeze/thaw cycles. Room temperature incubation led to a substantial increase of both plasma marker levels (Figure 2B).

**Figure 3:**
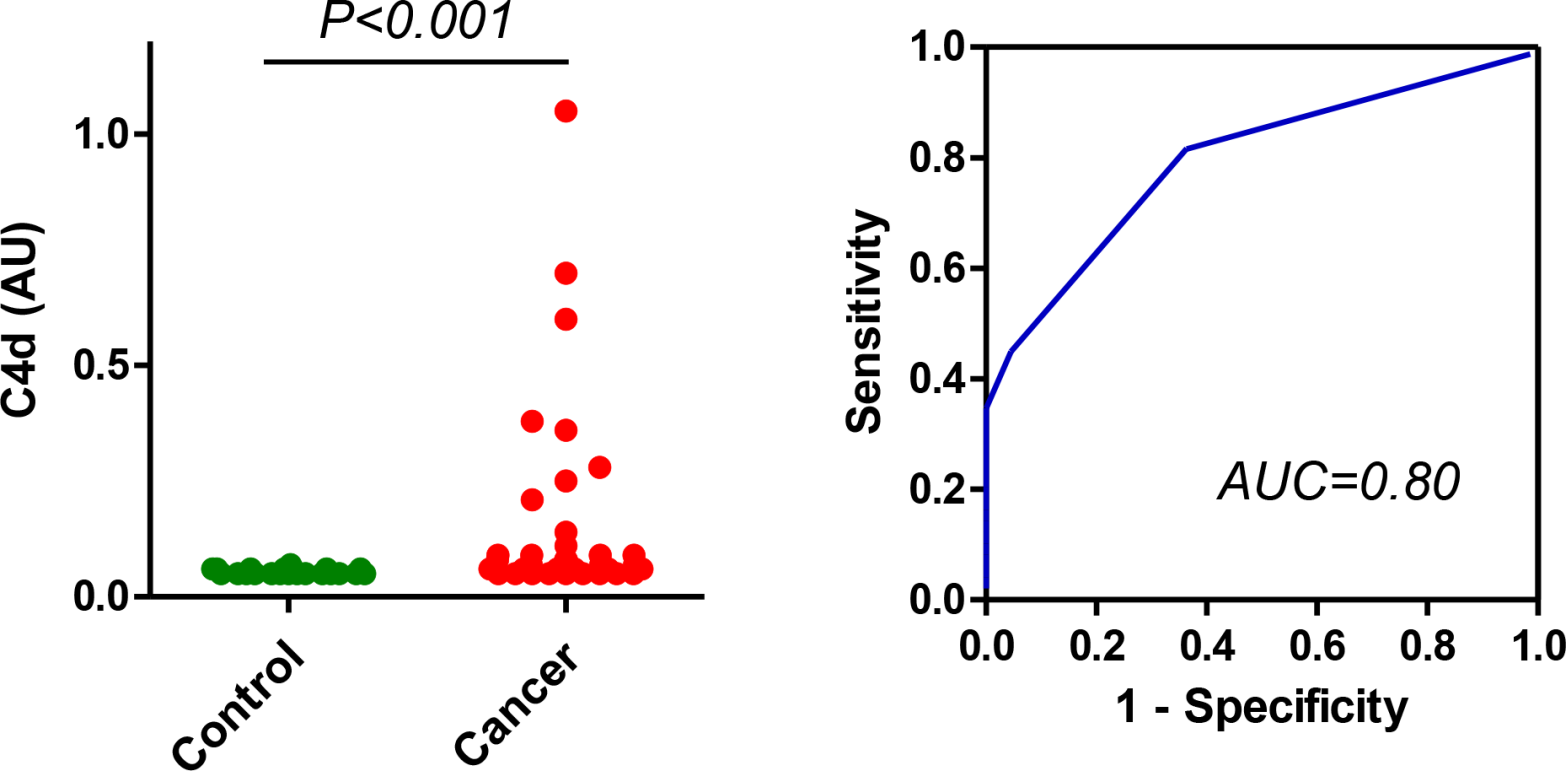
Quantitation of C4d in bronchoalveolar lavage samples from lung cancer patients and control subjects Levels of C4d in bronchoalveolar lavage supernatants from patients diagnosed with lung cancer and control subjects with non-malignant pulmonary diseases. The area under the curve was 0.80 (95% CI: 0.69–0.90). The *P* value was calculated using the two-sided Mann-Whitney *U*-test. AU: Arbitrary units.

We next explored the diagnostic potential of the specific measurement of C4d in bronchoalveolar lavage supernatants from 50 lung cancer patients and 22 patients with nonmalignant lung diseases obtained at the Clinica Universidad de Navarra. The levels of C4d-containing fragments have been previously reported in this cohort [15]. Briefly, these levels were 0.26 (0.11–0.53) µg/ml for lung cancer patients and 0.11 (0.11–0.22) µg/ml for control subjects (*P* < 0.001). The area under the ROC curve was 0.73 (95% CI = 0.61 to 0.84). The specific quantitation of C4d improved the performance of the assay (Figure 3). Of note, C4d could not be determined in one of the samples from the cancer group due to lack of material. C4d levels were 0.06 (0.06–0.09) AU for lung cancer patients and 0.05 (0.05–0.06) AU for control subjects (*P* < 0.001). The area under the ROC curve was 0.80 (95% CI = 0.69 to 0.90). No association was found between C4d levels and age, smoking status, histology or stage (Supplementary Table 2). A significant correlation was found between the levels of C4d-containing fragments and the levels of C4d (Spearman’s rho = 0.583; *P* < 0.001; Supplementary Figure 1B). These results strongly suggest that the assay based on specific antibodies reactive to C4d outperforms the assay based on antibodies against C4-derived fragments for the diagnosis of lung cancer.

### Quantitation of C4d for the assessment of lung cancer risk in asymptomatic individuals

The performance of the assays was then evaluated in patients enrolled in a lung cancer screening program. We used plasma samples from 20 CT-detected lung cancer patients from the Multicenter Italian Lung Detection (MILD) low-dose CT screening trial. Two plasma samples were available from each patient: a pre-diagnosis sample and a sample collected at surgery or at diagnosis. Each patient’s sample was matched to two samples from control individuals also included in the MILD trial. Table 1 summarizes the results. Neither the levels of C4d-containing fragments nor the specific levels of C4d were significantly associated with the risk of having lung cancer, although in the case of samples at diagnosis/surgery the association of the levels of C4d-containing fragments was closed to significance (odds ratio: 1.53; 95%CI: 0.93–2.51; *P* = 0.079). A significant correlation was found between the levels of C4d-containing fragments and the levels of C4d (Spearman’s rho = 0.274; *P* = 0.002; Supplementary Figure 1C).

**Table 1:** Association between risk of lung cancer and levels of C4d-containing fragments and C4d in plasma samples for the MILD screening program

Biomarker	Sample type	Odds ratio	95% CI	*P* value^*^
**C4d-containing fragments**	Pre-diagnosis	1.04	0.77–1.40	0.797
	At diagnosis/surgery	1.53	0.93–2.51	0.079
**C4d**	Pre-diagnosis	1.03	0.99–1.07	0.201
	At diagnosis/surgery	1.04	0.99–1.10	0.137

^*^Likelihood ratio test.

### Specific quantitation of C4d adds diagnostic value to the CT evaluation of indeterminate lung nodules

**Figure 4:**
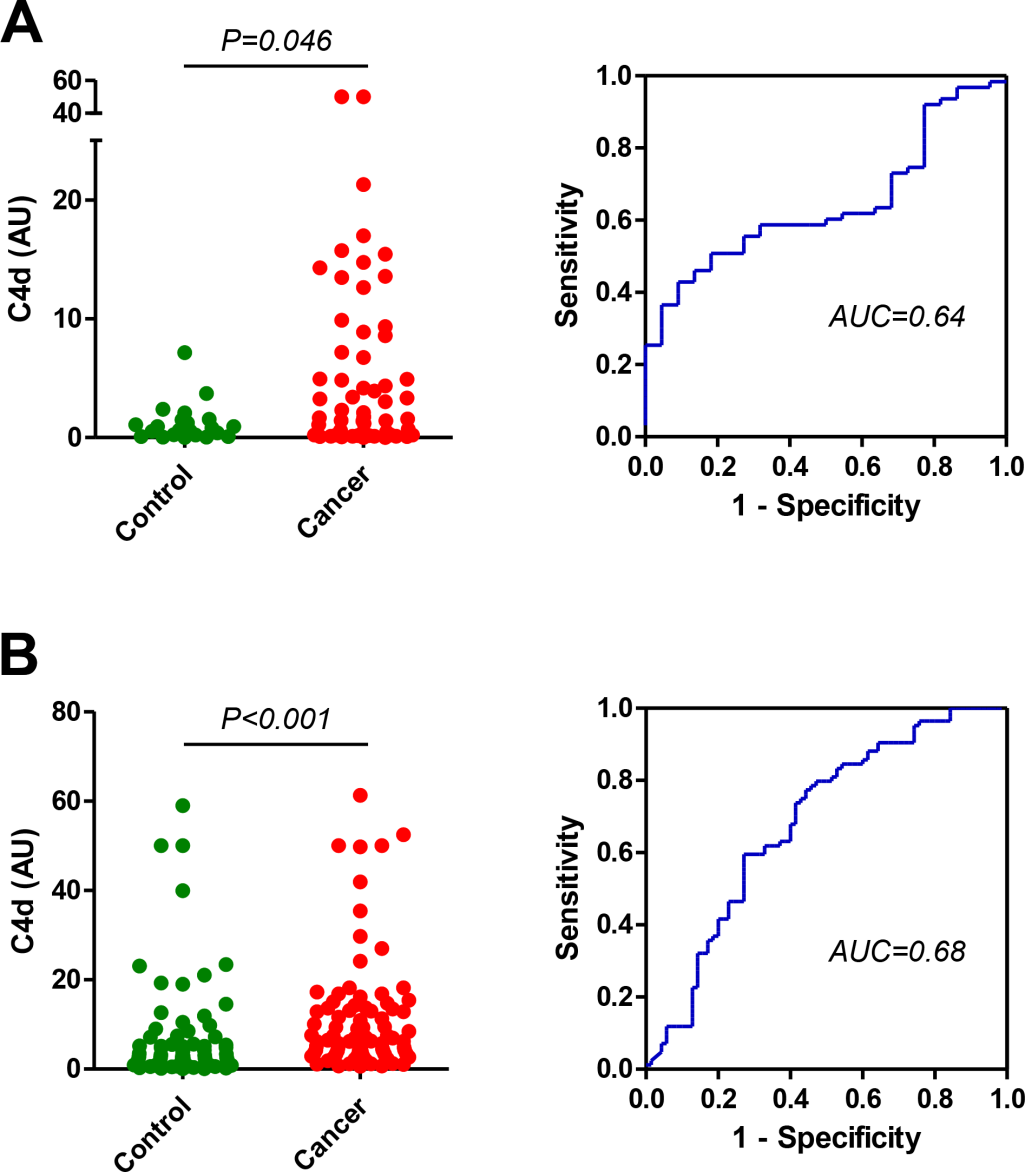
Quantitation of C4d in plasma samples from patients with indeterminate lung nodules (**A**) Plasma C4d levels and ROC curve in patients with indeterminate lung nodules that were diagnosed as lung cancer (*n* = 63) or no lung cancer (*n* = 22) (VUMC1 cohort). The area under the ROC curve was 0.64 (95% CI = 0.52 to 0.76). (**B**) Plasma C4d levels and ROC curve in patients with indeterminate lung nodules that were diagnosed as lung cancer (*n* = 84) or no lung cancer (*n* = 70) (VUMC2 cohort). The area under the ROC curve was 0.68 (95% CI = 0.60 to 0.77). *P* values were calculated using the two-sided Mann–Whitney *U*-test. AU: Arbitrary units.

We next evaluated the performance of the new antibodies in the diagnosis of indeterminate CT-detected lung nodules. We first used a cohort of plasma samples from 63 patients with lung cancer nodules and 22 patients with non-cancer nodules from Vanderbilt University Medical Center (VUMC1 cohort). Plasma samples from patients with malignant nodules presented significantly higher levels of C4d, as measured with the specific antibodies, than those from patients with benign nodules: 1.58 (7.17–6.94) vs 0.71 (0.23–1.48) AU (*P* = 0.046; Figure 4A). The area under the ROC curve was 0.64 (95% CI = 0.52 to 0.76). No association was found between C4d levels and epidemiological and clinical characteristics such as sex, age, tumor size, histology or stage (Supplementary Table 3). A significant association was observed with smoking status (*P* = 0.046) in the group of cancer patients, although no association was found with the number of pack-years.

The differences in C4d levels were validated in a larger cohort of plasma specimens from patients with indeterminate nodules consisting of 85 patients with lung cancer and 70 control patients from Vanderbilt University Medical Center (VUMC2 cohort). Plasma C4d levels were significantly increased in lung cancer patients when compared to their non-lung cancer counterparts: 6.72 (3.76–13.70) vs 3.08 (1.09–7.23) AU (*P* < 0.001; Figure 4B). The area under the ROC curve was 0.68 (95% CI = 0.60 to 0.77). Of note, C4d could not be determined in one cancer patient due to sample limitation. No significant associations were found with age, smoking status, tobacco consumption, tumor size or stage. Marker levels were significantly associated with sex in control subjects (*P* = 0.022) and with tumor histology in cancer patients (*P* = 0.020) (Supplementary Table 4). In support of the superiority of the specific determination of C4d, differences in plasma levels of C4d-containing fragments in patients with lung cancer and individuals without the disease did not reach statistical significance in any of the VUMC cohorts (Supplementary Figures 1D, 1E and 2).

Finally, to better assess the performance of the assay in a relevant clinical context, we selected patients from the VUMC2 cohort with nodules sizes ranging from 8 to 30 mm (35 controls and 32 cancers). This middle-sized pulmonary nodules show an intermediate risk of malignancy and pose a challenge to clinicians [20]. Statistically significant differences between cancer patients and controls were observed in this subgroup of patients: 6.34 (2.34–17.03) vs. 3.05 (1.48–8.95); *P* = 0.039. The prevalence of malignancy in this target population has previously been estimated as 23% [21]. Based on this estimated value, the test identified likely benign lung nodules with 84% negative predictive value and 54% positive predictive value, at 89% specificity and 44% sensitivity. Taken together, these results suggest that the specific determination of C4d adds diagnostic value to the CT evaluation of indeterminate lung nodules.

## DISCUSSION

In this study we demonstrate that the specific evaluation of C4d using newly developed antibodies improves the diagnostic performance of our previously proposed method based on the quantitation of C4d-containing fragments [15]. The superior diagnostic performance of the quantitation of C4d was observed in both plasma and bronchoalveolar lavage samples. Besides, it is important to mention that, although significant in most cases, the correlation between the two markers was surprisingly low. In particular, several samples showed high levels of C4d-containing fragments with low levels of C4d. These results suggest that the commercial assay detects certain C4d-containing fragments which are not detected by the assay that specifically determines C4d. This observation was already emphasized in the first study performed with the antibodies specific for the C4d neoepitope [19]. This previous work showed that the specific determination of C4d offered several advantages over the assay based on C4d-containg fragments. Firstly, the latter assay seemed to be dependent on changes in C4d tridimensional conformation, as evidenced by stability experiments with heat-inactivated or frozen samples in which the signal was greatly increased; secondly, external factors present *in vivo* may hinder or mimic conformational C4d neoepitopes; and thirdly, the antibodies against C4d-containing elements may recognize non-canonical fragments generated by unspecific proteolytic activities present in biological fluids. Based on our present data, we conclude that the recognition of these “extra fragments” does not add diagnostic value to the test, but may interfere with the precise determination of C4d, and may be more affected by stability issues.

Stability problems arising during the storage and management of biological materials are critical in evaluating their suitability for the quantification of biomarkers [22–23]. Stability studies in plasma samples from lung cancer patients confirmed the profound effect of freeze-thaw cycles over the quantitation of C4d-containing fragments [19], which is not observed when C4d is specifically determined with the antibodies against the C4d linear neoepitope resulting from specific cleavage. These observations suggest that this experimental manipulation leads to the generation of C4d-containing fragments by the action of unspecific factors that do not affect the canonical C4d linear neoepitope. On the other hand, the levels of both C4d-containing fragments and C4d were influenced by the exposition of the samples to prolong periods of incubation at room temperature. This could be due to ongoing spontaneous activation of complement under these conditions. This is highly relevant in the clinical routine, where proper handling procedures should be followed to maintain the integrity of the plasma and avoid distorted measurements. Other stability aspects, such as the influence of long-term frozen storage, should also be assessed.

To investigate the applicability of C4d quantification in the management of lung cancer, we evaluated the performance of this marker in two clinically relevant scenarios in which suitable biomarkers are urgently needed [24]: a better definition of high-risk individuals, and discrimination between benign and malignant nodules. Using samples from a subcohort of the international early lung cancer action project (I-ELCAP) carried out at Clinica Universidad de Navarra [25], we previously observed that the levels of C4d-containing fragments were associated with lung cancer risk in asymptomatic individuals [15]. In the present study we were unable to find significant differences in C4d levels between control and case samples from patients enrolled in the MILD trial [26]. In contrast, when we tested a cohort of samples from individuals with indeterminate pulmonary nodules, we obtained very encouraging results. In two independent cohorts of patients, the quantitation of C4d was able to discriminate between plasma samples from patients with lung cancer nodules and those from control subjects. Interestingly, the negative and positive predictive values of the test were comparable to a validated classifier applied in the same clinical setting [21].

There are limitations in this study that need to be addressed in the future. The quantitation assay for C4d is not standardized and the results are now expressed as arbitrary units. The undesirable consequence is that the ranges obtained from the different cohorts are too different, and at present no meaningful cut-off values can be established for future analyses. To optimize the use of the assay, it is imperative to develop standards and internal controls that allow an appropriate normalization. Only in that way, pre-established cut-off values may be determined and applied to definitively assess the performance of the assay. On the other hand, more detailed studies should be performed to evaluate the relationship of the marker with the clinicopathological characteristics of the patient, as well as with potential comorbidities that may interfere with the diagnosis of lung cancer. Finally, although the diagnostic yield of C4d for the classification of benign lung nodules is promising, we failed to validate the utility of the marker in the identification of individuals at risk of lung cancer. In a heterogeneous disease such as lung cancer, the analysis of a single biomarker is unlikely to be accurate enough to provide clinical benefit. Therefore, in order to enhance the diagnostic performance of the test and have an impact in the management of lung cancer patients, studies should be performed to develop classifiers in which the information provided by C4d can be combined with the information provided by other markers.

In conclusion, our study identifies C4d as a promising blood-based biomarker for the diagnosis of lung cancer, suggesting that its determination may serve as an adjunct to current clinical practice in the differentiation of indeterminate pulmonary nodules. Our results warrant the development of standardized assays and diagnostic panels based on the quantitation of this proteolytic product derived from complement activation.

## MATERIALS AND METHODS

### Clinical samples

The study included five independent clinical series, four of EDTA-plasma samples and one of bronchoalveolar lavage specimens. Cohorts and subgroups of patients were as follows. Plasma samples from Clinica Universidad de Navarra included specimens from 39 non-small cell lung cancer patients at stages I or II, and 39 control subjects. Demographics and clinical characteristics of these patients are shown in Supplementary Table 1. The series of bronchoalveolar lavage fluids from Clinica Universidad de Navarra included samples from 50 patients with lung malignancies and 22 patients with nonmalignant lung diseases. The procedure for bronchoalveolar lavage collection and the clinicopathological features of this cohort have been previously reported [27], and are summarized in Supplementary Table 2. Plasma samples from asymptomatic participants in the MILD screening trial included samples from 20 CT screen-detected lung cancer patients. Two plasma samples were available from each patient: a sample at pre-diagnosis (collected between 13 and 47 months prior to diagnosis or surgery) and a sample collected at surgery or at diagnosis. Each patient’s sample was matched to two control samples (matched by sex, age, smoking status, pack-years and collection date) from individuals included in the same screening program. Finally, two independent series obtained at Vanderbilt University Medical Center (VUMC) were also studied. Clinical features of these series are summarized in Supplementary Tables 3–5. The first one (VUMC1) included plasma samples from 85 patients presenting indeterminate lung nodules discovered by chest CT. Lung nodules were defined as rounded opacities completely surrounded by lung parenchyma. After pathological examination, 63 indeterminate lung nodules were diagnosed as lung cancers whereas the remaining 22 were diagnosed as non-malignant. The second series (VUMC2) included plasma samples from 85 patients with indeterminate lung nodules diagnosed as lung cancer and 70 patients with indeterminate lung nodules diagnosed as non-malignant. Diagnosis of non-malignant lung indeterminate nodules in VUCM1 and VUCM2 is indicated in Supplementary Table 5. Lung tumors were classified according to the WHO 2004 classification and the International System for Staging Lung Cancer [28–29]. All study protocols were carried out according to the Declaration of Helsinki, were approved by the Institutional Research Ethics Committees, and all patients gave informed consent.

### Biomarker measurements

Plasma samples were diluted 1:25 in PBS for the measurement of C4d-containing fragments and 1:50 for the specific measurement of C4d. Bronchoalveolar lavage specimens were diluted 1:10 for the analysis of both biomarkers. C4d-containing fragments were measured with C4d MicroVue ELISA (A008, Quidel) according to manufacturer’s instructions. This assay recognizes all C4d-containing fragments of activated C4, including C4b, iC4b and C4d. The specifically measurement of C4d was performed by means of an enzyme immunoassay based on a novel capture antibody, generated in one of our laboratories, against the linear neoepitope exposed at the C-terminus of human C4d [19]. Briefly, 96-well plates were coated with 3.5 µg/ml of this antibody. After blocking with 3% fish gelatin (Norland Products), diluted samples and standards were added to the plates. Standards consisted in serial dilutions of International Complement Standard #2 [30]. Detection was performed using a mouse anti-C4/C4d monoclonal antibody (#253; Quidel) diluted 1:1500, followed by incubation with goat anti-mouse HRP-conjugated antibody (Dako) diluted 1:1000. This ELISA recognizes C4d without cross-reactivity or interference with other C4d-containing fragments. All analyses were performed with blind-coded samples.

### Statistical analyses

Values are expressed as median (interquartile range). Normal distribution of the data was tested by the Shapiro-Wilk test. Differences between two or more groups were determined using the Mann-Whitney *U*-test or the Kruskal-Wallis test, respectively. Receiver-operating characteristic (ROC) curves were generated to evaluate the diagnostic accuracy of biomarkers. Ninety five percent confidence intervals (CI) were also calculated around the measurements. Conditional logistic regression was used to estimate odds ratios and 95% CI for lung cancer risk. Two-sided *P* values less than 0.05 were considered statistically significant. All statistical analyses were performed using Stata/IC 12.1.

## SUPPLEMENTARY MATERIALS FIGURES AND TABLES



## Abbreviations

AU: Arbitrary units
CI: confidence interval
CT: computed tomography
MILD: Multicenter Italian Lung Detection
VMUC: Vanderbilt University Medical Center
