# Supplementary material for: Complement C4d-specific antibodies for the diagnosis of lung cancer
Source: Oncotarget. 2017 Dec 26;9(5):6346–55. doi: 10.18632/oncotarget.23690 (PMC5814217; doi:10.18632/oncotarget.23690)
Supplement: Supplementary file 1 [file oncotarget-09-6346-s001.pdf]

# Complement C4d-specific antibodies for the diagnosis of lung cancer

## SUPPLEMENTARY MATERIALS

**Supplementary Table 1: Clinical characteristics and C4d plasma levels in non-small cell lung cancer patients and control individuals from Clinica Universidad de Navarra***

| Characteristics | *n* | Lung cancer patients | | *n* | Control subjects | |
|---|---|---|---|---|---|---|
| | | C4d (AU) | *P* value** | | C4d (AU) | *P* value** |
| **Sex** | | | | | | |
| Female | 5 | 1.04 (0.88–1.65) | 0.966 | 5 | 0.46 (0.39–0.77) | 0.585 |
| Male | 34 | 0.95 (0.69–1.61) | | 34 | 0.58 (0.52–0.65) | |
| **Age** | | | | | | |
| ≤65 | 19 | 0.89 (0.65–1.43) | 0.536 | 22 | 0.57 (0.46–0.61) | 0.336 |
| >65 | 20 | 1.02 (0.72–1.84) | | 17 | 0.59 (0.53–0.66) | |
| **Smoking status** | | | | | | |
| Never | 1 | 0.69 | 0.444 | | | |
| Former | 25 | 1.16 (0.70–2.06) | | 27 | 0.58 (0.52–0.66) | 0.287 |
| Current | 13 | 0.86 (0.78–1.08) | | 12 | 0.54 (0.44–0.63) | |
| **Pack-years** | | | | | | |
| ≤35 | 17 | 1.04 (0.80–1.43) | 0.472 | 18 | 0.59 (0.48–0.73) | 0.284 |
| >35 | 21 | 0.86 (0.68–1.68) | | 21 | 0.55 (0.52–0.61) | |
| Not available | 1 | 0.58 | | | | |
| **Histology** | | | | | | |
| ADC | 19 | 0.88 (0.69–2.06) | 0.757 | | | |
| SCC | 20 | 1.05 (0.70–1.52) | | | | |
| **Stage** | | | | | | |
| I | 31 | 0.88 (0.68–1.68) | 0.626 | | | |
| II | 8 | 1.10 (0.90–1.51) | | | | |

*Data are expressed as median (interquartile range); **Two-sided Mann-Whitney *U*-test.

**Supplementary Table 2: Clinical characteristics and C4d bronchoalveolar lavage levels in lung cancer patients from Clinica Universidad de Navarra***

| Characteristics | *n* | Lung cancer patients | | *n* | Control subjects | |
|---|---|---|---|---|---|---|
| | | C4d (AU) | *P* value** | | C4d (AU) | *P* value** |
| **Sex** | | | | | | |
| Female | 9 | 0.07 (0.06–0.09) | 0.326 | 8 | 0.06 (0.05–0.06) | 0.048 |
| Male | 40 | 0.06 (0.06–0.09) | | 14 | 0.05 (0.05–0.06) | |
| **Age** | | | | | | |
| ≤65 | 29 | 0.06 (0.06–0.09) | 0.807 | 14 | 0.05 (0.05–0.06) | 0.682 |
| >65 | 20 | 0.06 (0.06–0.11) | | 8 | 0.06 (0.05–0.06) | |
| **Smoking status** | | | | | | |
| Never | 7 | 0.06 (0.05–0.28) | 0.998 | 11 | 0.06 (0.05–0.06) | 0.088 |
| Former | 23 | 0.07 (0.05–0.14) | | 7 | 0.05 (0.05–0.05) | |
| Current | 18 | 0.06 (0.06–0.07) | | 4 | 0.05 (0.05–0.06) | |
| Not available | 1 | 0.05 | | | | |
| **Pack-years** | | | | | | |
| ≤35 | 19 | 0.06 (0.06–0.09) | 0.890 | 14 | 0.05 (0.05–0.06) | 0.263 |
| >35 | 26 | 0.06 (0.06–0.11) | | 7 | 0.05 (0.05–0.06) | |
| Not available | 4 | 0.07 (0.06–0.09) | | 1 | 0.05 | |
| **Histology** | | | | | | |
| ADC | 12 | 0.07 (0.06–0.09) | 0.910 | | | |
| SCC | 22 | 0.06 (0.06–0.11) | | | | |
| SCLC | 8 | 0.07 (0.06–0.11) | | | | |
| Other | 7 | 0.06 (0.05–0.06) | | | | |
| **Stage** | | | | | | |
| I-III | 15 | 0.06 (0.06–0.11) | 0.692 | | | |
| IV | 17 | 0.06 (0.05–0.07) | | | | |
| Not available | 17 | 0.07 (0.06–0.09) | | | | |

*Data are expressed as median (interquartile range); **Two-sided Mann-Whitney *U*-test (two-group comparisons), or two-sided Kruskal-Wallis *H* test (three-group comparisons).

**Supplementary Table 3: Clinical characteristics and C4d plasma levels in lung cancer patients from Vanderbilt University Medical Center (VUMC1 cohort)***

| Characteristics | *n* | Lung cancer patients | | *n* | Control subjects | |
|---|---|---|---|---|---|---|
| | | C4d (AU) | *P* value** | | C4d (AU) | *P* value** |
| **Sex** | | | | | | |
| Female | 23 | 1.41 (0.12–8.58) | 0.396 | 9 | 0.52 (0.1 -0.92) | 0.242 |
| Male | 40 | 1.83 (0.29–6.95) | | 13 | 0.93 (0.25–1.55) | |
| **Age** | | | | | | |
| ≤65 | 23 | 2.3 (0.24–6.73) | 0.850 | 10 | 0.46 (0.10–1.54) | 0.406 |
| >65 | 39 | 1.41 (0.22–8.87) | | 10 | 0.86 (0.54–1.48) | |
| Not available | 1 | 2.1 | | 2 | | |
| **Smoking status** | | | | | | |
| Never | 4 | 0.17 (0.06–0.28) | 0.046 | 6 | 0.86 (0.52–0.93) | 0.596 |
| Former | 47 | 2.09 (0.30–8.87) | | 11 | 0.54 (0.05–1.55) | |
| Current | 12 | 1.48 (0.22–5.32) | | 4 | 1.16 (0.24–4.60) | |
| **Pack-years** | | | | | | |
| ≤35 | 21 | 1.73 (0.21–9.35) | 0.878 | 11 | 0.93 (0.52–1.55) | 0.078 |
| >35 | 42 | 1.43 (0.24–6.73) | | 10 | 0.24 (0.05–0.61) | |
| Not available | 1 | 1.48 | | 1 | 1.48 | |
| **Tumor size** | | | | | | |
| ≤2 cm | 34 | 1.28 (0.28–4.82) | 0.488 | | | |
| >2 cm | 28 | 2.66 (0.22–8.02) | | | | |
| Not available | 1 | 3.24 | | | | |
| **Histology** | | | | | | |
| ADC | 34 | 1.51 (0.26–8.58) | 0.263 | | | |
| SCC | 25 | 1.09 (0.21–4.17) | | | | |
| Other | 4 | 5.25 (3.29–11.30) | | | | |
| **Stage** | | | | | | |
| IA | 39 | 1.58 (0.35–7.17) | 0.597 | | | |
| IB | 14 | 0.99 (0.17–4.91) | | | | |
| II | 3 | 0.28 (0.21–13.56) | | | | |
| III-IV | 3 | 9.88 (0.23–50) | | | | |
| Not available | 4 | 2.26 (0.60–3.62) | | | | |

[s]Data are expressed as median (interquartile range); **Two-sided Mann-Whitney *U*-test (two-group comparisons), or two-sided Kruskal-Wallis *H* test (comparisons of three or four groups).

**Supplementary Table 4: Clinical characteristics and C4d plasma levels in lung cancer patients from Vanderbilt University Medical Center (VUMC2 cohort)***

| Characteristics | *n* | Lung cancer patients | | *n* | Control subjects | |
|---|---|---|---|---|---|---|
| | | C4d (AU) Median (IQR) | *P* value* | | C4d (AU) Median (IQR) | *P* value* |
| **Sex** | | | | | | |
| Female | 28 | 10.31 (5.18–15.79) | 0.105 | 31 | 5.09 (0.97–19.02) | 0.022 |
| Male | 56 | 5.70 (3.35–12.98) | | 39 | 2.47 (1.09–5.04) | |
| **Age** | | | | | | |
| ≤65 | 37 | 5.14 (3.34–13.44) | 0.251 | 51 | 3.10 (1.24–7.23) | 0.522 |
| >65 | 47 | 7.43 (4.10–14.76) | | 19 | 3.05 (0.90–8.53) | |
| **Smoking status** | | | | | | |
| Never | 2 | 10.30 (7.48–13.12) | 0.659 | 17 | 3.94 (0.83–12.62) | 0.698 |
| Former | 46 | 7.27 (3.35–15.08) | | 28 | 3.26 (1.27–9.37) | |
| Current | 36 | 5.91 (3.85–13.20) | | 25 | 2.52 (1.35–5.21) | |
| **Pack-years** | | | | | | |
| ≤35 | 18 | 8.88 (6.48–13.12) | 0.414 | 36 | 2.97 (0.72–7.82) | 0.407 |
| >35 | 66 | 5.91 (3.35–14.28) | | 34 | 3.26 (1.66–7.23) | |
| **Tumor size** | | | | | | |
| ≤2 cm | 21 | 6.96 (2.24–14.77) | 0.836 | | | |
| >2 cm | 63 | 6.48 (4.04–13.12) | | | | |
| **Histology** | | | | | | |
| ADC | 28 | 12.05 (3.79–18.18) | 0.020 | | | |
| SCC | 19 | 4.04 (2.21–6.10) | | | | |
| LCC | 6 | 9.72 (8.44–12.96) | | | | |
| SCLC | 15 | 6.97 (4.20–13.72) | | | | |
| NSCLC NOS | 16 | 7.76 (4.29–13.55) | | | | |
| **Stage** | | | | | | |
| I-II | 23 | 6.20 (3.34–13.44) | 0.453 | | | |
| III | 25 | 10.08 (4.22–15.44) | | | | |
| IV | 15 | 4.75 (3.35–11.65) | | | | |
| Not available | 21 | 6.97 (4.20–13.72) | | | | |

*Data are expressed as median (interquartile range); **Two-sided Mann-Whitney *U* test (two-group comparisons), or two-sided Kruskal-Wallis *H* test (comparisons of three or more groups).

**Supplementary Table 5: Diagnosis of non-malignant lung indeterminate nodules in the two series from Vanderbilt University Medical Center (VUMC)**

**VUMC1**

| **Diagnosis** | ***n*** |
|---|---|
| Granulomatous lesion | 5 |
| Inflammation | 3 |
| COPD | – |
| Emphysema | 1 |
| Pneumonia | 1 |
| Hamartoma | 1 |
| Normal tissue | – |
| Others | 9 |
| Unknown | 3 |

**VUMC2**

| **Diagnosis** | ***n*** |
|---|---|
| Granulomatous lesion | 30 |
| Inflammation | 5 |
| COPD | 2 |
| Emphysema | 2 |
| Pneumonia | 2 |
| Hamartoma | 2 |
| Normal tissue | 6 |
| Others | 10 |
| Unknown | 13 |

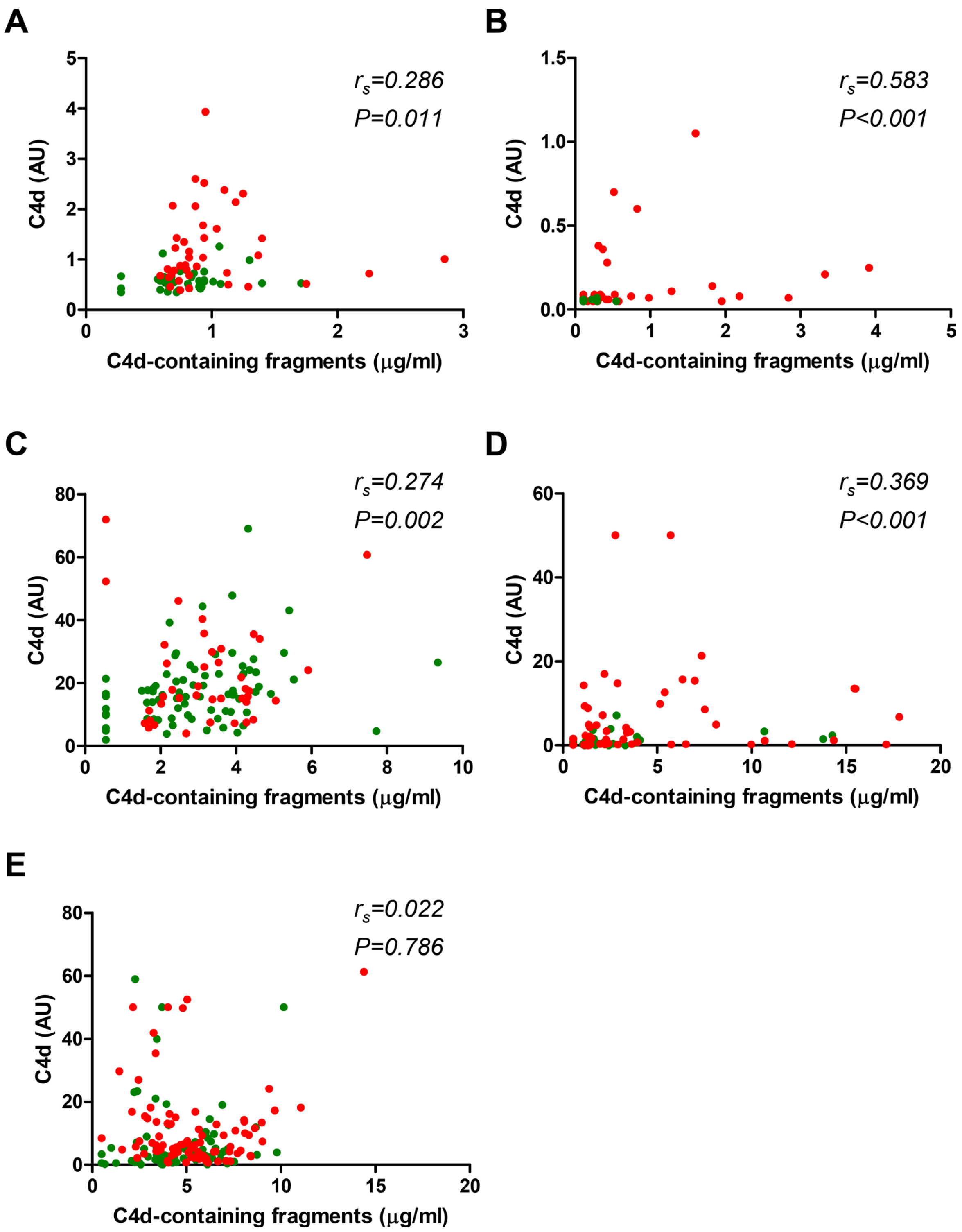


**Supplementary Figure 1:** Correlation between the levels of C4d-containing fragments and C4d in plasma samples from the CUN cohort (**A**), BAL samples from the CUN cohort (**B**), plasma samples from the MILD cohort (**C**), plasma samples from the VUMC1 cohort (**D**), and plasma samples from the VUMC2 cohort (**E**). Lung cancer cases are shown in red and control cases in green. Statistical significance of the correlations was analyzed using the Spearman rank test.

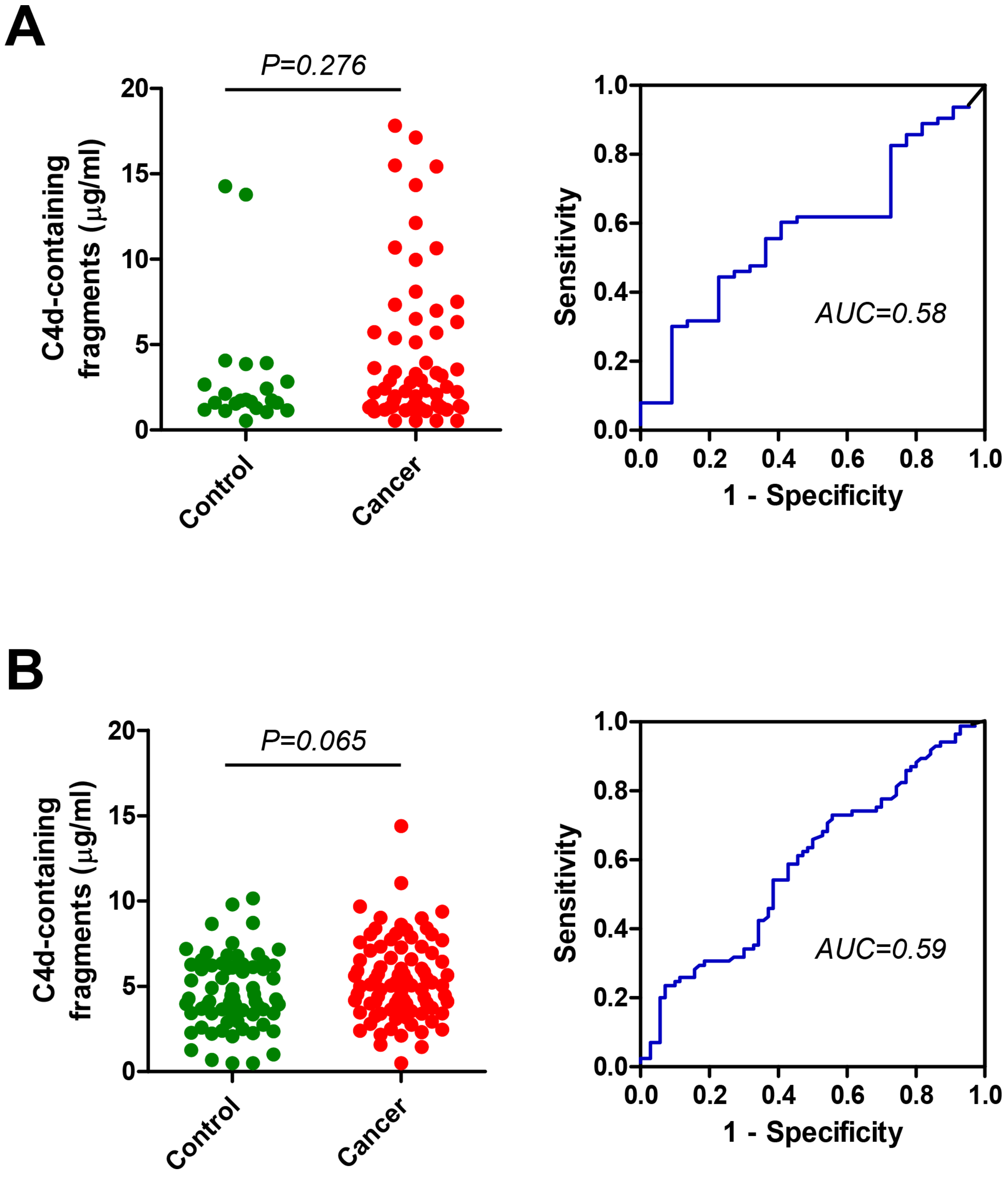


**Supplementary Figure 2: Quantitation of C4d-containing fragments in plasma samples from patients with indeterminate lung nodules in two independent series from the Vanderbilt University Medical Center (VUMC).** (**A**) Levels of plasma C4d-containg fragments and ROC curve in patients with indeterminate lung nodules diagnosed as lung cancer ($n$ = 63) or benign lesions ($n$ = 22) (VUMC1 cohort). The area under the ROC curve was 0.58 (95% CI = 0.45 to 0.71). (**B**) Plasma levels of C4d-containg fragments and ROC curve in patients with indeterminate lung nodules that were diagnosed as lung cancer ($n$ = 84) or benign lesions ($n$ = 70) (VUMC2 cohort). The area under the ROC curve was 0.59 (95% CI = 0.50 to 0.68). $P$ values were calculated using the two-sided Mann-Whitney $U$-test
